# Application of Spatial Methods in Rural Cancer Control Research in the United States

**DOI:** 10.1007/s40471-026-00380-2

**Published:** 2026-02-21

**Authors:** Whitney E. Zahnd, Jan M. Eberth, Sarah H. Nash, Amy L. Tran, Gabriel A. Benavidez, Michelle B. Shin, Malesa Pereira, Arrianna Marie Planey

**Affiliations:** 1 Department of Health Management and Policy, College of Public Health, University of Iowa, 145 N. Riverside Dr, Iowa City, IA 52242 USA; 2 Holden Comprehensive Cancer Center, University of Iowa, Iowa City, IA USA; 3 Department of Population Health Sciences, Virginia Tech, Blacksburg, VA USA; 4 Comprehensive Cancer Center, New Mexico Tumor Registry, Albuquerque, NM USA; 5 Division of Epidemiology, Biostatistics, and Preventive Medicine, Department of Internal Medicine, University of New Mexico, Albuquerque, NM USA; 6 Department of Health Promotion and Disease Prevention, Cancer Prevention and Control Research Network, University of North Carolina at Chapel Hill, Chapel Hill, NC USA; 7 Department of Public Health, Robbins College of Health and Human Sciences, Baylor University, Waco, TX USA; 8 Department of Child, Family, and Population Health Nursing, University of Washington, Seattle, WA USA; 9 Department of Community Health Sciences and Policy, LSU Health School of Public Health, New Orleans, LA USA; 10 Department of Health Policy and Management, University of North Carolina at Chapel Hill, Chapel Hill, NC USA; 11 Lineberger Comprehensive Cancer Center, University of North Carolina at Chapel Hill, Chapel Hill, NC USA

**Keywords:** Cancer, rural health, mapping and visualization, Spatial epidemiology, Spatial statistics

## Abstract

**Purpose of Review:**

The 66 million Americans who live in rural areas experience notable cancer disparities. It is imperative to examine the spatial elements of these disparities to know how to best target policies and interventions. Our review summarizes spatial methods in rural cancer control research.

**Recent Findings:**

Spatial methods relevant for rural cancer control research range from identifying patterns to spatial accessibility to regression approaches. Recent applications have adapted to the new rural health landscape by considering the role of telehealth in spatial accessibility measures and the impact of rural hospital closures in multilevel regression modeling with spatial considerations. Further, research continues to innovate on small area estimation techniques to provide useful risk factor and burden estimates to inform interventions.

**Summary:**

As rural cancer disparities persist, it is critical that researchers continue to develop and adapt spatial methods that will best inform policies and interventions to reduce these disparities.

## Introduction

As many as 66 million United States (U.S.) residents live in rural areas, comprising approximately 15–20% of the population; roughly 74–97% of U.S. land is geographically rural [1, 2] Compared to urban, rural populations experience notable differences and disparities across the cancer control continuum from etiology to mortality and survivorship [3]. This includes higher overall cancer incidence and mortality rates and site-specific differences for cancers amenable to prevention and screening opportunities (e.g., lung, colorectal, cervical, and human papillomavirus (HPV)-associated cancers) [4, 5]. Additionally, rural populations tend to be diagnosed with cancer at more advanced stages when treatments may be less effective [6]. High overall and late-stage cancer incidence may be due to lower rates of cancer screenings for cancer such as colorectal and cervical cancers [6, 7]. Similarly, lower rates of HPV vaccination among rural populations may play a role in the increasing rates of HPV-associated cancer [4, 8]. The consistently higher rates of smoking may also contribute to the higher rates of tobacco-associated cancers, broadly, and lung cancer specifically in rural communities [4, 9]. Rural populations also tend to have higher rates of uninsurance and worse spatial access to both primary and cancer-specific care, which may affect these populations’ ability to access and use prevention and screening services [10–12].

Addressing these disparities in rural cancer outcomes requires research methods that consider the dynamics of distance and isolation and their impacts across the cancer continuum. These methods must be applied appropriately, considering the limitations of administratively defined geographic units (e.g., counties, census tracts), varying rural definitions, and small numbers. An array of spatial methods can be used to examine access to cancer care, identify geographic cancer patterns, address small number concerns, and consider spatial dynamics using regression analyses. Our objective is to provide a summary of these methods and examples of how they have been applied in rural cancer control research.

### Defining “Rural”

Federal definitions of “rural” serve diverse programmatic, regulatory, and policy needs (Table 1). Frequently, “urban” or “metropolitan” is defined first, with areas not meeting these criteria being defined as “rural” or “non-metropolitan.” Rural-urban classifications are typically based on geographic units, such as counties, census tracts, or ZIP code tabulation areas (ZCTAs). Federal definitions of metropolitan or urban areas (and implicitly, non-metropolitan or rural areas) are updated in the years following a decennial census and reflect demographic shifts (population growth, in-migration) within and between geographies, as well as updates to the definitions themselves (e.g., considering population counts in definitions based on the 2010 census compared to definitions considering housing unit counts based on the 2020 census).

**Table 1:** Description of definitions used to characterize “rural” and “non-metropolitan” in cancer control research

Name	Originator	Geographic Unit	Definition of “rural” or “non-metropolitan”
Urban Areas/Urban Clusters (UA/UCs	U.S. Census Bureau	Census Block and Block Groups	Any area not defined as urban. Urban defined as having 425 housing units per square mile, and either at least 2,000 housing units, or a population of at least 5,000 people.
Core Based Statistical Areas (CBSAs)	U.S. Office of Management and Budget	County and County Equivalents	Areas outside of metropolitan areas (i.e., central or core counties with one or more urban areas of 50,000 or more persons as defined by the Census Bureau, and outlying counties that are economically tied to the core counties as measured by having 25% of workers living in the county commute to the central counties, or 25% of the employment in the county consists of workers coming out from the central counties).
Rural Urban Commuting Area (RUCA)	U.S. Department of Agriculture (USDA) Economic Research Service (ERS)	Census Tract, ZIP code approximation	RUCA’s primary codes classify Census tracts as 1–10 according to Census Bureau urban areas in combination with commuting information. RUCA codes 4 and above are typically considered non-metropolitan/rural.
Rural-Urban Continuum Codes (RUCC)	U.S. Department of Agriculture (USDA) Economic Research Service (ERS)	Counties and county equivalents	RUCC distinguishes U.S. metropolitan counties by the population size of their urban area (must be at least 5,000 people in a given area to be considered urban). Non-metropolitan counties are classified by their degree of urbanization and adjacency to a metropolitan area. RUCC classifies counties as 1–9, with 4 and above considered nonmetropolitan.
Frontier and Remote Area (FAR) Codes	U.S. Department of Agriculture (USDA) Economic Research Service (ERS)	0.5. x 0.5. kilometer grid cells, aggregated to ZCTAs	Frontier and remote aims to capture land characterized by low population and/or geographic remoteness. FAR areas are defined in relation to the time it takes to travel by car to the edges of nearby urban areas. FAR classifies areas as 1–4, with 4 being the most remote. Rural areas can be included in all four FAR codes.
Urban Influence Codes (UIC)	U.S. Department of Agriculture (USDA) Economic Research Service (ERS)	Counties and county equivalents	A 9-category classification based on the OMB Core Based Statistical Areas. Codes 4 and above are typically considered non-metropolitan/rural, with codes 4–6 representing small metropolitan and adjacent counties, and 7–9 representing counties not adjacent to metropolitan counties.
National Center for Health Statistics (NCHS)	Centers for Disease Control and Prevention (CDC) National Center for Health Statistics (NCHS)	Counties and county equivalents	A 6-category classification based on the OMB definition of urbanized areas. The NCHS rural-urban classification scheme defines urbanicity as a spectrum while defining non-metropolitan areas as either “micropolitan” or “non-core • The default rural-urban classification scheme for administrative datasets, such as BRFSS and NHIS

There is no single “gold standard” definition of rurality; however, several federal agencies and offices have created their own definitions. Many stem from the Office of Management and Budget’s (OMB) definition, which dichotomizes counties as metropolitan and non-metropolitan. This dichotomization is then used in definitions from the United States Department of Agriculture (USDA), and Centers for Disease Control and Prevention (CDC). Within dichotomous designations of rurality, geographies can be further classified by considering population size, proximity or adjacency to metropolitan, and commuting patterns. Choice of which definition to use and how these definitions are categorized in rural cancer control research varies and is dependent on the specific use case, including geographic level of data available and sample size. For example, studies of rare cancers may require analyses at coarser spatial resolutions due to small case counts at more local levels, and accordingly, rural definitions may be selected based on the spatial resolution of available data.

### Geospatial Analysis Methods Overview

#### Overview and Considerations

For this review, we consider components of cancer control as defined by the NCI’s cancer control continuum which includes etiology, prevention, screening, diagnosis, treatment, and survivorship [3]. We also consider surveillance measures like incidence, staging, and mortality, and measures of access to cancer-relevant health services. We describe the application of several types of spatial methods applied to rural cancer control research. These methods frequently use administrative units that can be categorized as rural. First, we describe methods that examine area-level, cancer-relevant measures such as mapping/visualization and identification of spatial clusters. Second, we describe methods used to characterize access to cancer care, ranging from distance calculations to more complex gravity-based model approaches. Finally, we discuss methods that integrate spatial elements into regression analyses, including spatial regression, hierarchical or multilevel models that consider spatial elements, and small-area estimation. Table 2 provides examples of studies that have used these methods across the cancer continuum.

**Table 2:** Spatial methods applied to rural cancer control across the continuum

	Etiology	Prevention	Screening and Detection	Incidence,Diagnosis, Staging	**Treatment**	**Mortality**
Identifying and Assessing Disease Patterns and Clusters	Kennedy C, Liu Y, Meng X, Strosnider H, Waller L, Zhou Y.Developing indices to identify hotspots of skin cancer vulnerability among the Non-Hispanic White population in the United States. Ann Epidemiol . 2021 Jul:59:64–71. doi: https://doi.org/10.1016/j.annepidem.2021.04.004. Epub 2021 Apr 22.	Ramphul *R*, Zamorano AS, Upadhyay S, Desai M, Bauer C. Spatiotemporal analysis of HPV vaccination and associated neighborhood-level disparities in Texas-an ecological study. Front Public Health. 2024 Jun 25:12:1418526. doi: https://doi.org/10.3389/fpubh.2024.1418526. eCollection 2024.	Zahnd WE, McLafferty SL, Sherman RL, Klonoff-Cohen, Farner S, Rosenblatt KA. Spatial Accessibility to Mammography Services in the Lower Mississippi Delta Region States. J Rural Health. 2019 Sep;35(4):550–559. doi: 10.1111/jrh.12349. Epub 2019 Jan 28.	Siegel SD, Zhang Y, Lynch SM, Rowland J, Curriero FC. A Novel Approach for Conducting a Catchment Area Analysis of Breast Cancer by Age and Stage for a Community Cancer Center. Cancer Epidemiol Biomarkers Prev . 2024 May 1;33(5):646–653. doi: 10.1158/1055-9965.EPI-23-1125.	Lin Y, Wimberly MC, Da Rosa *P*, Hoover J, Athas WF. Geographic access to radiation therapy facilities and disparities of early-stage breast cancer treatment. Geospat Health. 2018 May 7;13(1):622. doi: 10.4081/gh.2018.622.	Luo C, Khan S, Jin L, James AS, Colditz GA, Drake BF. Where Should the Cancer Control Interventions Target: A Geospatial Hotspot Analysis for Major Cancer Mortality 2018 to 2022 in the United States. Cancer Epidemiol Biomarkers Prev . 2025 Jul 1;34(7):1074–1079. doi: 10.1158/1055-9965.EPI-24-0957.
**Spatial Accessibility Measures**		Ranganathan R, Zahnd WE, Harrison SE, Brandt HM, Adams SA, Eberth JM. Spatial Access to Vaccines for Children Providers in South Carolina: Implications for HPV Vaccination. *Prev Chronic Dis* . 2020 Dec 24:17:E163. doi: 10.5888/pcd17.200300.	Anayanwu C, Yang K, Bonne V, Kalkbrenner AE, Kostelac C, Beyer KMM. Geographic access to lung cancer screening and environmental lung cancer risk factors in the contiguous United States. *Health Place*. 2025 Nov 27:97:103588. doi: https://doi.org/10.1016/j.healthplace.2025.103588. Online ahead of print.	Zahnd WE, Josey MJ, Schootman M, Eberth JM. Spatial accessibility to colonoscopy and its role in predicting late-stage colorectal cancer. *Health Serv Res*. 2021 Feb;56(1):73–83. doi: https://doi.org/10.1111/1475-6773.13562.13562. Epub 2020 Sep 20.	Liu X, Fluchell MN, Kirchoff AC, Zhu H, Onega T. Geographic Access to Pediatric Cancer Care in the US. *JAMA Netw Open* . 2023 Jan 3;6(1):e2251524. doi: https://doi.org/10.1001/jamanetworkopen.2022.51524.	
**Small area estimation techniques**	Eberth JM, McLain AC, Hong Y, Sercy E, Diedhiou A, Kilpatrick DJ. Estimating county-level tobacco use and exposure in South Carolina: a spatial model-based small area estimation approach. *Ann Epidemiol* . 2018 Jul;28(7):481–488.e4. doi: 10.1016/j.annepidem.2018.03.015. Epub 2018 Mar 30.	Eberth JM, Hossain MM, Tiro JA, Zhang X, Holt JB, Vernon SW. Human Papillomavirus Vaccine Coverage Among Females Aged 11 to 17 in Texas Counties: An Application of Multilevel, Small Area Estimation. *Womens Health Issues* . 2013 Mar-Apr;23(2):e131-41. doi: 10.1016/j.whi.2012.12.005.	Berkowitz Z, Zhang X, Richard TB, Sabatino S, Peipins LA, Holt J, White MC. Multilevel Regression for Small-Area Estimation of Mammography Use in the United States, 2014. *Cancer Epidemiol Biomarkers Prev* . 2019 Jan;28(1):32–40. doi: 10.1158/1055-9965.EPI-18-0367. Epub 2018 Oct 1.	Smith MJ, Roberts EK, Charlton ME, Oleson JJ. Incorporating small-area estimation into mediation analyses with areal datasets. *Spat Spatiotemporal Epidemiol* . 2025 Aug:54:100735. doi: 10.1016/j.sste.2025.100735. Epub 2025 Jul 21.		GBD US Health Disparities Collaborators. Burden of liver cancer mortality by county, race, and ethnicity in the USA, 2000-19: a systematic analysis of health disparities. *Lancet Public Health* . 2024 Mar;9(3):e186-e198. doi: 10.1016/S2468-2667(24)00002-1.
**Spatial Regression**	Boakye KA, Iyanda AE, Oppong JR, Lu Y. A multiscale analysis of social and spatial determinants of cancer and noncancer hazards from on-road air pollution in Texas. *Spat Spatiotemporal Epidemiol* . 2022 Jun:41:100484. doi: 10.1016/j.sste.2022.100484. Epub 2022 Jan 21.	Shreves AH, Buller ID, Chase E, Creutzfeldt H, Fisher JA, et al. Geographic Patterns in U.S. Lung Cancer Mortality and Cigarette Smoking. *Cancer Epidemiol Biomarkers Prev* . 2023 Feb 6;32(2):193–201. doi: 10.1158/1055-9965.EPI-22-0253.	Zahnd WE, McLafferty SL, Sherman RL, Klonoff-Cohen, Farner S, Rosenblatt KA. Spatial Accessibility to Mammography Services in the Lower Mississippi Delta Region States. *J Rural Health*. 2019 Sep;35(4):550–559. doi: 10.1111/jrh.12349. Epub 2019 Jan 28.	Muo L, Yang J, Deng G. Mapping rural-urban disparities in late-stage cancer with high-resolution rurality index and GWR. *Spat Spatiotemporal Epidemiol* . 2018 Aug:26:15–23. doi: 10.1016/j.sste.2018.04.001. Epub 2018 May 7.		Dong W, Bensken WP, Kim U, Rose J, Fan Q, Schiltz NK, Berger NA, Koroukian SM. Variation in and Factors Associated With US County-Level Cancer Mortality, 2008–2019
**Multilevel modeling with spatial consideration**	Goyal N, Camacho F, Mangano J, Goldenberg D. Evaluating for a geospatial relationship between radon levels and thyroid cancer in Pennsylvania. *Laryngoscope* . 2015 Jan;125(1):E45-9. doi: 10.1002/lary.24815. Epub 2014 Jul 7.	Wheeler DC, Miller CA, Do EK, Ksinan AJ, Trogdon JG et al. Identifying Area-Level Disparities in Human Papillomavirus Vaccination Coverage Using Geospatial Analysis. *Cancer Epidemiol Biomarkers Prev* (2021) 30 (9): 1689–1696. 10.1158/1055-9965.EPI-21-0331	Bauer C, Zhang K, Xiao Q, Lu J, Hong YR, Suk R. County-Level Social Vulnerability and Breast, Cervical, and Colorectal Cancer Screening Rates in the US, 2018. *JAMA Netw Open* . 2022 Sep 1;5(9):e2233429. doi: 10.1001/jamanetworkopen.2022.33429.	Snead R, Henry KA, Wilson RT, Schootman M, Jones RM. The effects of area-level deprivation on colorectal cancer incidence at the small area-level in Pennsylvania from 2008 to 2017. *Cancer Epidemiol* . 2025 Aug:97:102850. doi: 10.1016/j.canep.2025.102850. Epub 2025 May 31.	Ratnapridipa KL, Lian M, Jeffe DB, Davidson NO, Eberth JM, Pruitt S, Schootman M. Patient, Hospital, and Geographic Disparities in Laparoscopic Surgery Use Among Surveillance, Epidemiology, and End Results–Medicare Patients With Colon Cancer. *Diseases of the Colon & Rectum* 60(9):p 905–913, September 2017. | DOI: 10.1097/DCR.0000000000000874	Nash R, Switchenko JM, Ward KC, Collin LJ, Moubadder L, et al. A registry-based approach for estimating county-level race disparities in breast cancer mortality: an analysis in Georgia. *A J Epidemiol*, Volume 194, Issue 9, September 2025, Pages 2698–2704, 10.1093/aje/kwae413

#### Mapping and Visualization

Visualizing spatial patterns helps identify geographic disparities that may otherwise remain obscured in tabular data, allowing researchers, practitioners, and policymakers to identify high-burden areas, gaps in access to care, and opportunities for targeted intervention implementation and resource allocation [13]. This is especially important in rural areas, where populations are dispersed over large geographic areas, health infrastructure is limited, and cancer outcomes often vary across large distances [14]. Effective data visualization in the form of maps not only communicates these patterns clearly but also serves as the first step in guiding more advanced spatial analyses. Interactive, web-based maps and other visualization tools (e.g., StoryMaps) have increasingly been used by federal agencies (e.g., NCI’s State Cancer Profiles), states, and non-profit organizations to visualize county-level (e.g., rural relevant) data accessible [15, 16].

Spatial data are typically classified and displayed as points, lines, and/or polygons [17]. In rural cancer research, point data may represent the physical geographic location of an oncology clinic or screening facility; lines can be used to represent transportation networks that impede or facilitate physical access to care between patients and healthcare providers; whereas polygons represent administrative geographic boundaries such as counties, census tracts, or health service areas, which are useful for aggregating area-level data (e.g., cancer incidence). One of the most commonly used visualization approaches in cancer research is the choropleth map, which uses color patterns and shading gradients to represent variation in a given variable across geographic regions [18]. Traditional choropleth maps visualize one variable, while bivariate choropleth maps apply a combined grouping scheme to two variables to depict both simultaneously and identify geographic areas with particular value combinations (e.g., high–high, low–low). For example, areas with low cervical cancer screening rates and limited access to the Breast and Cervical Cancer Early Detection Program may represent important targets for intervention [19, 20]. Choropleth maps are the most widely used because they provide an intuitive overview of spatial patterns. However, their use in rural contexts requires caution. Large rural counties can mask within-county heterogeneity and give the false impression of uniformity across the entire geographic area [21]. This is true for geographically large counties defined as urban with notable rural areas, such as Coconino County Arizona containing both the isolated Grand Canyon and the urban, Flagstaff, Arizona, and geographically large rural counties pervasive in the U.S. West. It is important to only display standardized numbers (e.g., rates or percentages) in choropleth maps and to interpret these maps as reflecting land area not population.

Despite the usefulness of mapping rural cancer data, there are also inherent challenges. Small population sizes often lead to unstable rates requiring smoothing or empirical Bayes techniques to avoid misleading patterns [22]. For example, Ward and colleagues used these approaches to calculate incidence and mortality rates and related relative risks across ZCTAs [23]. Data suppression rules designed to protect privacy and prevent misinterpretation can introduce missingness to maps of cancer outcomes, often excluding rural communities and obscuring cancer burden in sparsely populated areas. Additionally, the modifiable areal unit problem (MAUP), which includes both aggregation (scale) and grouping (zonal) effects, can influence how cancer outcomes appear depending on the geographic units used for analysis [24]. The aggregation effect occurs when associations observed at larger geographic units (i.e., counties) do not persist when analyses are conducted at smaller geographic units (i.e., census blocks).

#### Identifying and Assessing Disease Patterns and Clusters

Simple and bivariate choropleth maps are useful for visualizing spatial patterns but cannot determine whether observed patterns (e.g., spatial clustering or apparent correlations between variables) are statistically significant. To assess statistical significance, Local Indicators of Spatial Association (LISA) methods are required, including Local Moran’s I for identifying spatial clusters and outliers or Getis–Ord Gi* statistic for detecting hot and cold spots [25]. These approaches extend beyond descriptive mapping by integrating statistical inference directly into spatial visualizations. They retain the intuitive display of spatial patterns while adding statistical measures that help distinguish meaningful spatial structure from patterns likely due to chance. LISA-based approaches have been applied in rural cancer control research, for example, to examine spatial patterns in cancer surveillance outcomes (e.g., incidence) and access to cancer screening at the county level [26, 27].

Another commonly used approach for identifying disease clusters is Kernel Density Estimation (KDE), a spatial filtering method that transforms vector data (e.g., point or polygon input data) to generate raster density surface. While KDE is typically applied to more densely populated urban settings, methodological adaptations have been applied to address population density and spatial heterogeneity. These adaptations reduce over-smoothing in densely populated areas while stabilizing estimates in rural settings, allowing neighborhood-level variation to be visualized while minimizing spatial aggregation bias. For example, Carlos and colleagues identified meaningful population-level variation in alcohol outlet exposure that was not apparent using a static KDE approach [28].

No single method adequately addresses both population density and heterogeneity to produce highly reliable results. Goldberg and Cockburn illustrate how variation in geocoding practices can affect cancer surveillance estimates, depending on the geographic level at which cases are geocoded [29]. Simulation-based approaches, as proposed by Luo et al. and applied to breast cancer staging, offer a strategy for addressing aggregation effects by explicitly incorporating within-area heterogeneity [30]. Nonparametric spatio-temporal methods and KDE offer adaptive frameworks for identifying disease hotspots across space and time. Collectively, these approaches highlight the value of integrating multiple GIS methods to generate high-quality, interpretable results and more accurately characterize disease burden within and across rural populations.

#### Spatial Accessibility Measures

Many definitions and measures of healthcare accessibility are inclusive of multiple dimensions of access, including but not limited to, availability, accessibility, accommodation, affordability, and acceptability—collectively known as the “5 A’s of Access” [31, 32]. Conceptually, these dimensions are categorized as either spatial (availability and accessibility) or aspatial (accommodation, affordability, and acceptability) [32]. *The spatial dime*nsions include *availability*,* which* refers to the supply of services (e.g., number of oncologists in a community), and *accessibility*, which considers transportation-related factors and associated costs, typically measured as travel impedance, travel times, or other travel-related costs associated with healthcare access and use. Healthcare accessibility is often studied in terms of *potential* and *realized* access [33]. Potential access refers to measures of potential costs/burden of accessing existing healthcare services, and realized access refers to the actual uptake or utilization of healthcare services.

There are multiple ways to conceptualize and measure spatial accessibility of healthcare, including container methods, measures of travel impedance or distance, and gravity model-based measures of spatial accessibility (see Table 3). The simplest measure of availability is a container approach that enumerates the number of providers or facilities within an administrative boundary like a county, Census tract, or ZCTA. The supply-to-demand ratio (i.e., provider-to-population ratio), also a container approach, improves upon simple area-level counts because the denominator accounts for the population in need of the services being enumerated. However, container approaches are limited by their reliance on fixed administrative unit boundaries and the assumptions that all persons within an administrative unit have equal access to services, and that people do not seek care outside of the area defined by the container [34]. Using GIS, one can calculate distance-based measures of *potential access* (e.g., the minimum distance between origin points (e.g., patient address or residential ZIP code centroid, or population-weighted centroid and one or facilities) or *realized access* (i.e., actual travel distances and/or times based on the facility where patients received care).These approaches are particularly meaningful for rural cancer control research and have been used to examine access to healthy foods, cancer screening services, clinical trials and many other cancer-relevant services [35–38].

**Table 3:** Common spatial methods used to measure access to care in cancer control & outcomes research

Spatial Method	Input Data Types	Assumptions	Limitations
Travel distance or time	Points	• Patient travel directly from home (or centroid of census tract or other geographic unit) for care • Travel time calculations assume researcher specified time of travel, traffic patterns, and speed limits	• Depending on the choice of distance measure (e.g., Euclidian vs. Network) and assumed mode of transportation, this approach may underestimate or overestimate actual spatial accessibility of services for specific patient populations • Data that enable researchers to know the distance between patient residence and site of care often do not contain data about patients’ transportation mode; therefore, this may underestimate travel costs for people who do not own a personal vehicle or rely on public transportation or Medicaid-administered transportation benefits.
Voronoi or Theissen Polygons	Point, Polygon, Line	*Euclidian distance captures spatial extent & magnitude of environmental exposure; or range of activity space or facility service areas • Like other container methods, there is an implicit assumption that people only use healthcare services within the administrative areal unit they live in.	· Typically based on Euclidian distance · Creates a single, discrete “service area” for every point · Implicit assumption: no competition; no bypass on the part of patients · Providers have multiple practice locations: You’ll see this when you handle administrative datasets- there are unique ID numbers for providers and for facilities
Buffers	Point, Polygon, Line	Euclidian distance captures spatial extent & magnitude of environmental exposure; or range of activity space or facility service areas	· Typically based on Euclidian distance · Unless the distance thresholds are theoretically grounded (e.g., differential mobility of older or disabled patients), this can be an arbitrary measure
Standard Deviational Ellipse (SDE)	Point	Descriptive global measure of point distribution, represents dispersion and orientation of points in a spatial dataset	SDE output is affected by the number of points in the spatial dataset, and the size and shape of study area
Kernel Density Estimation (KDE)	Point	Euclidian distance is a suitable measure of proximity · If the KDE method does not account for road networks (i.e., it is based on Euclidian distance), it may underestimate the actual frictions of distance borne by patients	KDE output is sensitive to · Bandwidth parameters (fixed or adaptive) · Selection of kernel function (can proxy for distance decay function) · Size and shape of study area · Edge effects, also termed “spatial censoring”
2-Step Floating Catchment Area (2SFCA) Method & Gravity-Based Model Variants	Point and Polygon	A “spatial decomposition” method (Luo & Wang, 2003) that is a special case of a gravity model that accounts for demand · Defines spatial accessibility within distance-based catchment areas · Allows for competition between providers or facilities · Accounts for facility or provider capacity · Distance decay functions can be theoretically justified based on the population and services of interest	· The output values (e.g., spatial accessibility index) are relative measures that are not generalizable · Arbitrary selection of distance thresholds (based on 15-minute increments) · Dichotomous measure of spatial accessibility; implicit interpretation: all locations outside of the catchment have no access at all · Earlier variants of FCA do not account for travel impedance within catchment areas · Distance decay function is often assumed to be linear when it can be exponential

Gravity models that account for the interaction between supply and demand locations are an improvement over container methods. These models specify an impedance value, which quantifies the difficulty or cost to traverse between two points. Estimation of the impedance value (i.e., distance decay function) can be regionally specific and vary by mode of transportation and the type of service being considered. Similarly, the investigator must decide on the appropriate administrative areal units (e.g., ZCTAs, Census blocks or tracts) on which to base the model. Floating catchment area (FCA) methods are a type of gravity model developed to address the problem of using arbitrary administrative boundaries to assess the regional availability of services and to more realistically model cross-border interaction. The most basic FCA method involves calculating the provider-to-population ratio within a specified radius or travel time of a population location (e.g., Census tract centroid). Luo and Wang applied this method to study primary care physician shortage designations in northern Illinois using Census population data [39]. They later developed the two-step FCA (2SFCA) method, which uses floating catchment areas vs. fixed administrative boundaries to measure provider-to-population ratios [40]. The 2SFCA method has been improved and modified several times since its initial publication in 2003, resulting in the three-step floating catchment area (3SFCA), enhanced two-step floating catchment area (E2SFCA), integrated floating catchment area (iFCA), modified two-step floating catchment area (M2SFCA), and variable two-step floating catchment area (V2SFCA) approaches [41–45]. Zahnd et al. compared three of these methods (2SFCA, E2SFCA and V2SFCA) in the context of assessing the relationship between spatial accessibility to colonoscopy in South Carolina and stage at diagnosis [46]. Further, in a literature review examining spatial accessibility and breast cancer, Khan-Gates et al. found that increased access to mammography was associated with increased utilization of screening and/or decreased likelihood of late-stage diagnosis in some, but not all, of the studies examined [47]. Studies that examined time and distance to mammography were less likely to find correlations with breast cancer outcomes, while gravity or density-based scoring approaches were more likely to find significant correlations. More recent innovations of the 2SFCA method have considered not just physical location of providers, but also the impact of broadband access through a two-step virtual catchment area (2SVCA) [48, 49]. With the increasing role of telehealth in cancer care, considering both spatial and virtual access to care is critical.

#### Small Area Estimation Techniques

The size of the population of interest or the sample selected for surveillance efforts, including large nationally representative surveys used to measure cancer-relevant population health (e.g., Behavioral Risk Factor Surveillance System, Health Information National Trends Survey), directly impacts the feasibility and quality of derived estimates. Areas with small sample sizes may yield imprecise estimates with wide confidence intervals due to greater uncertainty in estimates. Conversely, larger sample sizes generally produce more precise estimates with narrower confidence intervals. In contrast, central cancer registries—key sources of post-diagnosis cancer data in the U.S.—collect information on all diagnosed cancer cases within a defined geographic area. Nevertheless, registry-based analyses can encounter small numbers, for example, when examining rare cancers or minoritized populations in rural settings, where both case counts and underlying population denominators may be small, or in some instances, zero.

As researchers seek to understand more local dynamics, they may wish to create estimates for areas with small populations (e.g., rural communities and tightly defined neighborhoods). Researchers have harnessed synthetic or indirect estimation methods to generate small area estimates, which are particularly helpful for estimating cancer incidence, cancer screening prevalence, and other cancer-relevant measures in rural counties or smaller geographic units with limited sample sizes [50]. These methods leverage information from areas with sufficient samples to predict outcomes in areas with insufficient, or in some cases, zero samples. These methods, however, are not without limitations. Zgodic et al. describes how specific areas can have wider predicted confidence intervals than expected due to the underlying demographic distribution of residents and associated model fixed effects [51]. In that study, more homogeneity in the residents of an area was associated with better model prediction, while the model underperformed in areas with greater demographic diversity of its residents. Due to the unique nature of their populations and lack of geographic adjacency to other US states, Alaska and Hawaii may also underperform statistically in such models. It is important, therefore, to examine the margins of error associated with estimates derived from these methods.

Small area estimation techniques have been applied to produce, cancer-relevant public databases such as the 500 Cities Project (2016–2019), CDC PLACES (2020–2024), and NCI State Cancer Profiles data [52, 53]. Small area estimation techniques must be used when an area sample is too small to yield direct estimates with adequate precision and reliability; thus, these methods employ statistical models to provide estimates for the target survey population and major population subgroups in the small area. Both the 500 Cities Project and CDC PLACES employed a validated multilevel regression and poststratification technique, which involves constructing a multilevel logistic regression model for each measure of interest. Independent variables are selected based on model performance and may include individual-level characteristics, such as age, sex, race/ethnicity, and educational attainment from the BRFSS, as well as county-level percentage of adults below 150% of the federal poverty level from the American Community Survey (ACS) 5-year estimates [50, 54]. Models are fitted with state- and county-level random effects, applying the final model to census block-level population counts to compute a predicted probability, then using Monte Carlo simulation to generate 1,000 simulated datasets for the point estimate and 95% confidence intervals. NCI State Cancer Profiles uses a Bayesian hierarchical model, including weighted survey data from the BRFSS, NHIS, and demographic variables from the ACS to generate model-based small area estimates at the Census tract and ZCTA levels [55].

Small area estimation approaches may also be applied to spatially aggregated administrative healthcare claims and cancer registry data. For example, a recent study leveraged bespoke geographies (created using the intersection of counties and ZIP codes) to enable spatial aggregation of healthcare claims and cancer registry data, applying Bayesian Conditional Autoregressive (CAR) logistic models with 10,000 MCMC simulations to estimate the prevalence of being up-to-date with colorectal cancer (CRC) screening among screen-eligible adults in North Carolina in the year 2020 [56]. Applying these methods to population-based survey, surveillance, and claims data can help provide important estimates for small populations, such as rural counties and small geographies to information public health interventions.

#### Spatial Regression

Spatial regression modeling is useful for analyzing multivariable relationships among spatially distributed dependent (e.g., cancer rates or access to care measures) and independent variables (e.g., sociodemographic characteristics). These approaches have the advantage of accounting for spatial autocorrelation, which is critical because spatial data often violate assumptions of independence (that observed values at one location are unrelated to those at another) and stationarity (that model properties do not vary by location or direction). To the latter point, in spatially referenced data, covariance depends on distance and direction, *not* absolute location. Therefore, for most spatial regression models, a prerequisite step is selecting and defining a spatial weights matrix (also termed “spatial weight”), which represents the spatial structure of the data by defining the “neighbors” for each areal unit in the study area. Spatial regression models can be characterized as global or local. Global model outputs include single parameters for each variable in the model (e.g., spatial lag or spatial error models). Local models allow parameters to vary locally. In the case of Geographically-Weighted Regression (GWR) and Multi-scale Geographically-Weighted Regression (MGWR) models, the output parameters incorporate effect sizes of varying bandwidths. In rural cancer research, spatial regression analyses are typically conducted at the county or census tract level. For example, one study used spatial lag models to assess sociodemographic factors associated with census tract-level access to mammography services within the largely rural Delta Regional Authority [27]. GWR methods have been used, for example, to examine local associations between provider density and prostate cancer mortality across rural and urban counties, as well as factors associated with breast cancer reconstruction within a single state [57, 58].

#### Multilevel Modeling with Spatial Considerations

Multilevel (or hierarchical) modeling approaches have been frequently used to consider both individual-level and area-level (macro-level) factors and their association with cancer across the continuum [59]. The spatial resolution of analysis— either meso (e.g., census tracts) or macro (e.g., counties)— will be driven by data availability considerations. For rural-focused analysis, this typically includes macro-level factors. Multilevel modeling has been characterized as an “ideal statistical approach” for rural cancer outcomes research [60]. These approaches account for the non-independence of observations within groups (e.g., cases aggregated to geographic units), subsequently improving the accuracy of standard error calculations and reducing Type I errors [61]. Multilevel modeling approaches where individuals are nested in counties or other geographies without explicitly considering spatial elements may not fully demonstrate associations between rurality and other measures and cancer outcomes. Spatial considerations in multilevel modeling approaches can be considered in a few ways, including employing a Bayesian approach to the multilevel models or cross-classified models (e.g., models in which individual are simultaneously nested in counties and hospitals) or including important spatially derived factors in multilevel regression analyses [62]. For example, a 2024 study applied mixed-effects generalized linear models to examine cancer screening uptake among screen-eligible rural-dwelling adults, adjusting for exposure to rural hospital closures, spatial accessibility to acute care, and the local supply of specialists [63]. This example underscores the importance of accounting for changes in the rural healthcare landscape, such as the impact of rural hospital closures, across the cancer continuum.

## Conclusions

As rural cancer disparities persist, it is imperative that epidemiologic, biostatistical, and health services research methods account for rurality not only as a categorical construct, but also as a spatially dynamic one. This includes the use of mapping and other spatial analytic approaches that explicitly consider distance, adjacency, and the challenges posed by small numbers. In parallel, spatial accessibility measures must continue to innovate to reflect changing patterns of care-seeking, including shifts in travel behavior and the growing use of telehealth. Finally, regression-based approaches—including model-based small area estimation, spatial regression, and multilevel modeling—are critical for informing interventions and policy decisions and for improving understanding of how changes to the rural health care landscape, such as rural hospital closures, shape cancer outcomes across the continuum.

##  Key References

- Sahar L, Foster SL, Sherman RL, Henry KA, Goldberg DW, Stinchomb DG, Bauer JE. GIScience and cancer: State of the art and trends for cancer surveillance and epidemiology. *Cancer*. 2019 Aug 1;125(15):2544-2560. doi: 10.1002/cncr.32052. Epub 2019 May 30.
  - **○ **This paper provides an important introduction to spatial methods, including data management elements like geocoding, and mapping best practices applied to cancer surveillance.
- Zahnd WE, McLafferty SL, Eberth JM. Multilevel analysis in rural cancer control: A conceptual framework and methodological implications. *Prev Med.* 2019 Sep 11;129 Suppl:105835. doi: 10.1016/j.ypmed.2019.105835.
  - **○ **This paper provides a conceptual framework for multilevel analysis applied to rural cancer control research as well as suggestions for building on these methods to improve applications.
- Planey AM, Wong S, Planey DA, Winata F, Ko MJ. Longer travel times to acute hospitals are associated with lower likelihood of cancer screening receipt among rural-dwelling adults in the U.S. South. *Cancer Causes Control*. 2025 Mar;36(3):297-308. doi: 10.1007/s10552-024-01940-x. Epub 2024 Nov 22.
  - **○ **This paper provides an example of the use of CDC Places data (derived using small area estimation techniques), distance to care measures, and policy relevant variables like Medicaid expansion status within multilevel regression models applied to cancer screening data.
- Liu L, Onega T, Moen EL, Tosteson A, Smith RE, Wang Q, Cowan L, Wang F. Digital divides in telehealth accessibility for cancer care in the United States. *NPJ Digit Med*. 2025 Aug 19;8(1):534. doi: 10.1038/s41746-025-01931-5.
  - **○ **This study innovates on traditional 2SFCA methods by considering virtual access to care that reflects telehealth accessibility for cancer care, as telehealth remains an important conduit for cancer care and amid persistent inequities in broadband access.

## Data Availability

No datasets were generated or analysed during the current study.

## References

[1] How We Define Rural. | HRSA [Internet]. [cited 2025 Dec 17]. https://www.hrsa.gov/rural-health/about-us/what-is-rural. Accessed 17 Dec 2025.

[2] Farrigan T, Genetin B, Sanders A, Pender J, Thomas KL, Winkler R, Cromartie J. Rural America at a glance: 2024 edition. Report No. EIB-282. U.S. Department of Agriculture, Economic Research Service; 2024.

[3] Cancer Control Continuum | Division of Cancer Control and Population Sciences. (DCCPS) [Internet]. [cited 2025 Dec 19]. https://cancercontrol.cancer.gov/about-dccps/about-cc/cancer-control-continuum. Accessed 19 Dec 2025.

[4] Semprini J, Gadag K, Williams G, Muldrow A, Zahnd WE. Rural-urban cancer incidence and trends in the united States, 2000 to 2019. Cancer Epidemiol Biomarkers Prev. 2024;33:1012–22. 10.1158/1055-9965.EPI-24-0072. 38801414 10.1158/1055-9965.EPI-24-0072

[5] Sokale IO, Raza SA, Thrift AP. Disparities in cancer mortality patterns: a comprehensive examination of U.S. rural and urban adults, 1999–2020. Cancer Med. 2023;12:18988–98. 10.1002/cam4.6451. 37559501 10.1002/cam4.6451 PMC10557857

[6] Zahnd WE, Fogleman AJ, Jenkins WD. Rural-urban disparities in stage of diagnosis among cancers with preventive opportunities. Am J Prev Med. 2018;54:688–98. 10.1016/j.amepre.2018.01.021. 29550163 10.1016/j.amepre.2018.01.021

[7] Benavidez GA, Sedani AE, Felder TM, Asare M, Rogers CR. Rural-urban disparities and trends in cancer screening: an analysis of Behavioral Risk Factor Surveillance System data (2018–2022). JNCI Cancer Spectr. 2024;8:pkae113. 10.1093/jncics/pkae113. 39520403 10.1093/jncics/pkae113 PMC11671142

[8] Walker TY, Elam-Evans LD, Williams CL, Fredua B, Yankey D, Markowitz LE, et al. Trends in human papillomavirus (HPV) vaccination initiation among adolescents aged 13–17 by metropolitan statistical area (MSA) status, National Immunization Survey - Teen, 2013–2017. Hum Vaccin Immunother. 2020;16:554–61. 10.1080/21645515.2019.1671765. 31662024 10.1080/21645515.2019.1671765 PMC7227639

[9] Erath TG, Chen FF, DeSarno M, Higgins ST. Examining US adolescent cigarette smoking prevalence by rurality and gender, 2002–2019. Prev Med. 2025;201:108377. 10.1016/j.ypmed.2025.108377. 40716718 10.1016/j.ypmed.2025.108377 PMC12366418

[10] Eisenstein S, Huang X, McBride T, Mueller K. Health insurance coverage in rural. and Urban Areas in the U.S.; 2023.

[11] Onega T, Alford-Teaster J, Wang F. Population-based geographic access to parent and satellite National cancer Institute cancer center facilities. Cancer. 2017;123:3305–11. 10.1002/cncr.30727. 28464212 10.1002/cncr.30727

[12] Zhang D, Son H, Shen Y, Chen Z, Rajbhandari-Thapa J, Li Y, et al. Assessment of changes in rural and urban primary care workforce in the United States from 2009 to 2017. JAMA Netw Open. 2020;3:e2022914. 10.1001/jamanetworkopen.2020.22914. 33112401 10.1001/jamanetworkopen.2020.22914 PMC7593812

[13] Nikolova S, Aleksandrova T. Geospatial insights into healthcare accessibility in Europe: a scoping review of GIS applications. Healthcare. 2025;13:2865. 10.3390/healthcare13222865. 41302253 10.3390/healthcare13222865 PMC12652348

[14] Bhatia S, Landier W, Paskett ED, Peters KB, Merrill JK, Phillips J, et al. Rural-Urban Disparities in Cancer Outcomes: Opportunities for Future Research. J Natl Cancer Inst. 2022;114:940–52. 10.1093/jnci/djac030. 35148389 10.1093/jnci/djac030 PMC9275775

[15] State Cancer Profiles [Internet]. [cited 2025 Dec 19]. https://statecancerprofiles.cancer.gov/. Accessed 19 Dec 2025.

[16] Rural-Urban Patterns of Cancer [Internet]. ArcGIS StoryMaps. 2025 [cited 2025 Dec 19]. https://storymaps.arcgis.com/stories/945bfe5c58f6472fa75e5d75cdde571a. Accessed 19 Dec 2025.

[17] Cromley E, McLafferty SL. GIS and Public Health. 2nd ed. Guilford Press; 2012.

[18] Schiewe J. Empirical studies on the visual perception of spatial patterns in choropleth maps. KN - Journal of Cartography and Geographic Information. 2019;69:217–28. 10.1007/s42489-019-00026-y.

[19] Biesecker C, Zahnd WE, Brandt HM, Adams SA, Eberth JM. A bivariate mapping tutorial for cancer control resource allocation decisions and interventions. Prev Chronic Dis. 2020;17:E01. 10.5888/pcd17.190254. 31895673 10.5888/pcd17.190254 PMC6977777

[20] Sahar L, Foster SL, Sherman RL, Henry KA, Goldberg DW, Stinchcomb DG, et al. GIScience and cancer: state of the art and trends for cancer surveillance and epidemiology. Cancer. 2019;125:2544–60. 10.1002/cncr.32052. 31145834 10.1002/cncr.32052 PMC6625915

[21] Bennett KJ, Borders TF, Holmes GM, Kozhimannil KB, Ziller E. What is rural? Challenges and implications of definitions that inadequately encompass rural people and places. Health Aff Proj Hope. 2019;38:1985–92. 10.1377/hlthaff.2019.00910. 10.1377/hlthaff.2019.00910 31794304

[22] Goovaerts P. Accounting for rate instability and spatial patterns in the boundary analysis of cancer mortality maps. Environ Ecol Stat. 2008;15:421–46. 10.1007/s10651-007-0064-6. 19023455 10.1007/s10651-007-0064-6 PMC2136438

[23] Ward C, Oleson J, Jones K, Charlton M. Showcasing cancer incidence and mortality in rural ZCTAs using risk probabilities via spatio-temporal bayesian disease mapping. Appl Spat Anal Policy. 2019;12:907–21. 10.1007/s12061-018-9276-4.

[24] Fontanet CP, Carlos H, Weiss JE, Diaz MCG, Shi X, Onega T, et al. Evaluating geographic health disparities in cancer care: example of the modifiable areal unit problem. Ann Surg Oncol. 2023;30:6987–9. 10.1245/s10434-023-14140-9. 37658267 10.1245/s10434-023-14140-9 PMC11166173

[25] Shekhar S, Evans MR, Kang JM, Mohan P. Identifying patterns in spatial information: a survey of methods. WIREs Data Min Knowl Discov. 2011;1:193–214. 10.1002/widm.25.

[26] Siegel RL, Sahar L, Robbins A, Jemal A. Where can colorectal cancer screening interventions have the most impact? Cancer Epidemiol Biomarkers Prev. 2015;24:1151–6. 10.1158/1055-9965.EPI-15-0082. 26156973 10.1158/1055-9965.EPI-15-0082

[27] Zahnd WE, McLafferty SL, Sherman RL, Klonoff-Cohen H, Farner S, Rosenblatt KA. Spatial accessibility to mammography services in the lower Mississippi delta region States. J Rural Health. 2019;35:550–9. 10.1111/jrh.12349. 10.1111/jrh.12349 30690797

[28] Carlos HA, Shi X, Sargent J, Tanski S, Berke EM. Density estimation and adaptive bandwidths: a primer for public health practitioners. Int J Health Geogr. 2010;9:39. 10.1186/1476-072X-9-39. 20653969 10.1186/1476-072X-9-39 PMC2920858

[29] Goldberg DW, Cockburn MG. The effect of administrative boundaries and geocoding error on cancer rates in California. Spat Spatio-Temporal Epidemiol. 2012;3:39–54. 10.1016/j.sste.2012.02.005. 10.1016/j.sste.2012.02.005 PMC3324674 22469490

[30] Luo L, McLafferty S, Wang F. Analyzing spatial aggregation error in statistical models of late-stage cancer risk: a Monte Carlo simulation approach. Int J Health Geogr. 2010;9:51. 10.1186/1476-072X-9-51. 20959015 10.1186/1476-072X-9-51 PMC2970586

[31] Levesque J-F, Harris MF, Russell G. Patient-centred access to health care: conceptualising access at the interface of health systems and populations. Int J Equity Health. 2013;12:18. 10.1186/1475-9276-12-18. 23496984 10.1186/1475-9276-12-18 PMC3610159

[32] Penchansky R, Thomas JW. The concept of access: definition and relationship to consumer satisfaction. Med Care. 1981;19:127–40. 10.1097/00005650-198102000-00001. 7206846 10.1097/00005650-198102000-00001

[33] Khan AA, Bhardwaj SM. Access to health care. A conceptual framework and its relevance to health care planning. Eval Health Prof. 1994;17:60–76. 10.1177/016327879401700104. 10132481 10.1177/016327879401700104

[34] Guagliardo MF. Spatial accessibility of primary care: concepts, methods and challenges. Int J Health Geogr. 2004;3:3. 10.1186/1476-072X-3-3. 14987337 10.1186/1476-072X-3-3 PMC394340

[35] Agunwamba AA, Zhu X, Sauver JS, Thompson G, Helmueller L, Finney Rutten LJ. Barriers and facilitators of colorectal cancer screening using the 5As framework: a systematic review of US studies. Prev Med Rep. 2023;35:102353. 10.1016/j.pmedr.2023.102353. 37576848 10.1016/j.pmedr.2023.102353 PMC10415795

[36] Raber M, Jackson A, Basen-Engquist K, Bradley C, Chambers S, Gany FM, et al. Food insecurity among people with cancer: nutritional needs as an essential component of care. J Natl Cancer Inst. 2022;114:1577–83. 10.1093/jnci/djac135. 36130287 10.1093/jnci/djac135 PMC9745434

[37] Tini G, Trapani D, Duso BA, Beria P, Curigliano G, Pelicci PG, et al. Quantifying geographical accessibility to cancer clinical trials in different income landscapes. ESMO Open. 2022;7:100515. 10.1016/j.esmoop.2022.100515. 35738201 10.1016/j.esmoop.2022.100515 PMC9271515

[38] McDonald YJ, Goldberg DW, Scarinci IC, Castle PE, Cuzick J, Robertson M, et al. Health service accessibility and risk in cervical cancer prevention: comparing rural versus nonrural residence in New Mexico. J Rural Health. 2017;33:382–92. 10.1111/jrh.12202. 27557124 10.1111/jrh.12202 PMC5939944

[39] Luo W, Wang F. Measures of spatial accessibility to healthcare in a GIS environment: synthesis and a case study in Chicago region. Environ Plan B Plan Des. 2003;30:865–84. 10.1068/b29120. 10.1068/b29120 PMC8238135 34188345

[40] Wang F, Luo W. Assessing spatial and nonspatial factors for healthcare access: towards an integrated approach to defining health professional shortage areas. Health Place. 2005;11:131–46. 10.1016/j.healthplace.2004.02.003. 15629681 10.1016/j.healthplace.2004.02.003

[41] Wan N, Zou B, Sternberg T. A three-step floating catchment area method for analyzing spatial access to health services. Int J Geogr Inf Sci Taylor Francis. 2012;26:1073–89. 10.1080/13658816.2011.624987.

[42] Luo W, Qi Y. An enhanced two-step floating catchment area (E2SFCA) method for measuring spatial accessibility to primary care physicians. Health Place. 2009;15:1100–7. 10.1016/j.healthplace.2009.06.002. 19576837 10.1016/j.healthplace.2009.06.002

[43] Bauer J, Groneberg DA, Public Library of Science. Measuring spatial accessibility of health care providers – introduction of a variable distance decay function within the floating catchment area (FCA) method. PLoS One. 2016;11:e0159148. 10.1371/journal.pone.0159148. 27391649 10.1371/journal.pone.0159148 PMC4938577

[44] Delamater PL. Spatial accessibility in suboptimally configured health care systems: a modified two-step floating catchment area (M2SFCA) metric. Health Place. 2013;24:30–43. 10.1016/j.healthplace.2013.07.012. 24021921 10.1016/j.healthplace.2013.07.012

[45] Luo W, Whippo T. Variable catchment sizes for the two-step floating catchment area (2SFCA) method. Health Place. 2012;18:789–95. 10.1016/j.healthplace.2012.04.002. 22560115 10.1016/j.healthplace.2012.04.002

[46] Zahnd WE, Josey MJ, Schootman M, Eberth JM. Spatial accessibility to colonoscopy and its role in predicting late-stage colorectal cancer. Health Serv Res. 2021;56:73–83. 10.1111/1475-6773.13562. 32954527 10.1111/1475-6773.13562 PMC7839638

[47] Khan-Gates JA, Ersek JL, Eberth JM, Adams SA, Pruitt SL. Geographic access to mammography and its relationship to breast cancer screening and stage at diagnosis: a systematic review. Womens Health Issues Off Publ Jacobs Inst Womens Health. 2015;25:482–93. 10.1016/j.whi.2015.05.010. 10.1016/j.whi.2015.05.010 PMC4933961 26219677

[48] Liu L, Onega T, Moen EL, Tosteson ANA, Smith RE, Wang Q, et al. Digital divides in telehealth accessibility for cancer care in the United States. NPJ Digit Med. 2025;8:534. 10.1038/s41746-025-01931-5. 40830581 10.1038/s41746-025-01931-5 PMC12365046

[49] Alford-Teaster J, Wang F, Tosteson ANA, Onega T. Incorporating broadband durability in measuring geographic access to health care in the era of telehealth: a case example of the 2-step virtual catchment area (2SVCA) method. J Am Med Inform Assoc. 2021;28:2526–30. 10.1093/jamia/ocab149. 34414437 10.1093/jamia/ocab149 PMC8510332

[50] Kong AY, Zhang X. The use of small area estimates in place-based health research. Am J Public Health. 2020;110:829–32. 10.2105/AJPH.2020.305611. 32298183 10.2105/AJPH.2020.305611 PMC7204458

[51] Zgodic A, Eberth JM, Breneman CB, Wende ME, Kaczynski AT, Liese AD, et al. Estimates of childhood overweight and obesity at the region, state, and county levels: a multilevel small-area estimation approach. Am J Epidemiol. 2021;190:2618–29. 10.1093/aje/kwab176. 34132329 10.1093/aje/kwab176 PMC8796862

[52] Limitations & Uses for the Model-based Small Area Estimates of Cancer Risk Factors and Screening Behaviors. - Small Area Estimates | SRP/DCCPS/NCI/NIH [Internet]. [cited 2025 Dec 17]. https://sae.cancer.gov/nhis-brfss/limitations.html. Accessed 17 Dec 2025.

[53] CDC. PLACES: Local Data for Better Health [Internet]. PLACES Local Data Better Health. 2025 [cited 2025 Dec 17]. https://www.cdc.gov/places/index.html. Accessed 17 Dec 2025.

[54] Zhang X, Holt JB, Yun S, Lu H, Greenlund KJ, Croft JB. Validation of multilevel regression and poststratification methodology for small area estimation of health indicators from the behavioral risk factor surveillance system. Am J Epidemiol. 2015;182:127–37. 10.1093/aje/kwv002. 25957312 10.1093/aje/kwv002 PMC4554328

[55] Liu B, Parsons V, Feuer EJ, Pan Q, Town M, Raghunathan TE, et al. Small area estimation of cancer risk factors and screening behaviors in US counties by combining two large national health surveys. Prev Chronic Dis. 2019;16:E119. 10.5888/pcd16.190013. 31469068 10.5888/pcd16.190013 PMC6716412

[56] Reeder-Hayes KE, Jackson BE, Reich BJ, Kuo T-M, Laurie E, Lund JL, et al. Statewide surveillance of colorectal cancer screening using geographic and insurance claims data. JCO Oncol Pract. 2025;21:162–162. 10.1200/OP.2025.21.10_suppl.162.

[57] Yao N, Foltz SM, Odisho AY, Wheeler DC, Public Library of Science. Geographic analysis of urologist density and prostate cancer mortality in the United States. PLoS One. 2015;10:e0131578. 10.1371/journal.pone.0131578. 26110832 10.1371/journal.pone.0131578 PMC4482500

[58] Liston JM, Samuel A, Camacho TF, Anderson RT, Campbell CA, Stranix JT. The state of breast cancer reconstruction in Virginia: an evidence-based framework for identifying locoregional health disparities. Ann Plast Surg. 2022;89:365–72. 10.1097/SAP.0000000000003276. 36149976 10.1097/SAP.0000000000003276

[59] Zahnd WE, McLafferty SL. Contextual effects and cancer outcomes in the United States: a systematic review of characteristics in multilevel analyses. Ann Epidemiol. 2017;27:739-748.e3. 10.1016/j.annepidem.2017.10.002. 29173579 10.1016/j.annepidem.2017.10.002

[60] Meilleur A, Subramanian SV, Plascak JJ, Fisher JL, Paskett ED, Lamont EB. Rural residence and cancer outcomes in the United States: issues and challenges. Cancer Epidemiol Biomarkers Prev. 2013;22:1657–67. 10.1158/1055-9965.EPI-13-0404. 24097195 10.1158/1055-9965.EPI-13-0404 PMC3814162

[61] Diez-Roux AV. Multilevel analysis in public health research. Annu Rev Public Health. 2000;21:171–92. 10.1146/annurev.publhealth.21.1.171. 10884951 10.1146/annurev.publhealth.21.1.171

[62] Ratnapradipa KL, Lian M, Jeffe DB, Davidson NO, Eberth JM, Pruitt SL, et al. Patient, hospital, and geographic disparities in laparoscopic surgery use among Surveillance, Epidemiology, and End Results-Medicare patients with colon cancer. Dis Colon Rectum. 2017;60:905–13. 10.1097/DCR.0000000000000874. 28796728 10.1097/DCR.0000000000000874 PMC5643006

[63] Planey AM, Wong S, Planey DA, Winata F, Ko MJ. Longer travel times to acute hospitals are associated with lower likelihood of cancer screening receipt among rural-dwelling adults in the U.S. south. Cancer Causes Control. 2025;36:297–308. 10.1007/s10552-024-01940-x. 39576391 10.1007/s10552-024-01940-x

